# Pneumonia

**Published:** 1864-01

**Authors:** 


*Pneumonia.*—This disease has prevailed in the city to a considerable extent during the last two months. The sudden fatal termination of several cases at near the same time, in families of extensive acquaintance, led the public to believe that a new or unusual epidemic had appeared, which defied medical treatment. There is reason to suppose that treatment is sometimes too much influenced by the name of the disease. And in none, perhaps, is the error more pernicious than in pneumonia. When the symptoms and signs lead to the diagnosis of inflammation of the lungs—the mind at once reverts to the principles of treatment applicable to the phlegmasia, and Venesection, Tartar emetic, Calomel, &c., are at once likely to be applied with an activity equaled to the intensity of the attack. The physician who has seen much of this disease in its adynamic type, will be at no loss to predict the result of such practice.

It has been our fortune to meet with a number of these cases recently, in all stages, from the first onset to symptoms indicative of impending collapse, and we have seldom been more forcibly impressed with the efficacy of the *sustaining* treatment than in this disease during the present season.

There is little room to doubt that the fear of tonics and stimulants in the early stage of inflammation, has allowed many patients to sink too low with Typhoid Pneumonia to be restored by any means. With us the conviction is firmly fixed, that the idea of *antiphlogistics* in the treatment of this form of pneumonia cannot be banished too soon from the mind.

We have in more than one instance seen the dry skin become relaxed and moist—the quick, hard pulse become slow and soft—the hurried and difficult respiration become free and regular—the dusky hue of countenance disappear and the clouded intellect become clear nnder the influence of Quinine, Opium, stimulants and a nutritious diet.

Two grains of Quinine with five grains Dover's Powders may be given every four hours—varying the dose, to meet the emergency of the particular case—after the first day or two stimulants may be added in quantity proportioned to the tendency to prostration of the vital forces of the system, and their effect upon the patient. When the surface becomes a little relaxed and the patient suffers pain then a blister over the seat of pain will afford manifest relief.

This is a hasty outline of the plan which with us has demonstrated that this serious disease which tends to rapid prostration, is amenable to treatment. We are forced to the conclusion that the physician will succeed best by omitting depressing agents, and not fearing to use sustaining remedies even in the early stage.

